# Heavy menstrual bleeding diagnosis and medical management

**DOI:** 10.1186/s40834-017-0047-4

**Published:** 2017-07-24

**Authors:** Intira Sriprasert, Tarita Pakrashi, Thomas Kimble, David F. Archer

**Affiliations:** 1 Department of Obstetrics and Gynecology, Faculty of Medicine, Chiang Mai University, Chiang Mai, Thailand; 2 Department of Obstetrics and Gynecology, Jones Institute for Reproductive Medicine/Eastern Virginia Medical School, Norfolk, VA USA; 3 CONRAD Clinical Research Center, Department of Obstetrics and Gynecology, Eastern Virginia Medical School, Norfolk, VA USA

**Keywords:** Heavy menstrual bleeding, Abnormal uterine bleeding, Tranexamic acid, Combination hormonal contraceptives, Non-steroidal anti-inflammatory drugs, Danazol

## Abstract

Heavy menstrual bleeding (HMB) is a common gynecological problem that has a significant impact on a woman’s quality of life and the activities of daily living. Due to the difficulty in accurately describing menstrual bleeding abnormalities using older terminology, the PALM-COEIN classification system of the Federation Internationale de Gynecologie et d’Obstetrique was proposed to describe and identify the etiology of abnormal endometrial bleeding. As there is no single pathway that is associated with HMB, there are several therapeutic interventions involving different molecular pathways to reduce HMB. This article will highlight the current evidence as it relates to the etiology of HMB as well as medical modalities of treatment.

## Background

Heavy menstrual bleeding (HMB) has been arbitrarily defined as a menstrual blood loss (MBL) of 80 ml or greater in both research [1] and clinical [2] settings. The National Institute for Health and Care Excellence in the United Kingdom proposed a definition of HMB as an excessive menstrual blood loss that interferes with the woman’s physical, emotional, social and material quality of life and this can occur alone or in combination with other symptoms and with a menstrual blood loss of <80 mL [3]. This definition is useful for the woman who does not meet the standard blood loss criteria for HMB but menstrual blood loss has a significant impact on her quality of life.

Measuring blood loss in clinical trials requires collecting all sanitary pads and tampons used during menstruation, and extracting the hemoglobin using the alkaline hematin method to estimate blood loss [4]. Collecting and storing used sanitary pads and tampons is often difficult and impractical for many women. An alternative is the pictorial blood loss assessment chart (PBAC) that visually estimates the extent of blood on sanitary pads and tampons [5]. Total menstrual fluid loss by weighting sanitary products before and after use is a reasonably accurate estimation of blood loss [6]. The weight increase on the used pad/tampon reflects total fluid loss which has been correlated with blood loss measured using the alkaline hematin method [6]. Blood contributed 50% of the total volume of menstrual fluid loss in women with heavy menstrual bleeding [6]. These methods, although more practical do not correlate with women’s experience of HMB and bothersome periods but provide a fairly reliable estimate of menstrual blood loss [6, 7].

Heavy menstrual bleeding is one of the most common gynecological problems, which accounts for 18–30% of gynecologic visit [8–10] and results in 17.8 surgical procedure per 10,000 reproductive age women in United States [11]. HMB has significant impact on a women’s physical, psychological, social, professional and family perspectives along with loss of work due to inability to leave home due to the amount of blood loss, decreased work productivity due to frequent changes of pads and tampons, and limited social activities with fear of embarrassment because of soiling outer garments with blood [12].

A large cohort study evaluating the healthcare resources used, lost work productivity, direct and indirect costs, and treatment patterns associated with HMB reported high rates of surgical intervention and increased healthcare resource utilization along with the costs [10]. Their estimated annual direct cost associated with HMB was 1 billion dollars while the indirect costs could be as high as 12 billion dollars because of the impact on work (days lost) and quality of life for the woman [12]. These figures do not account for intangible costs and productivity loss due to absenteeism. Iron deficiency anemia with its symptoms of fatigue, weakness, pallor, and dizziness is a major coexisting medical problem. The all cause costs for women with HMB and anemia is higher than for women with HMB alone [13].

HMB could be treated with both medical and surgical interventions and both methods are safe, acceptable and effective. Although hysterectomy is a definitive treatment for heavy menstrual bleeding with or without other gynecologic conditions such as fibroids, adenomyosis or endometriosis. a medical treatment is the preferred primary intervention in most circumstances as surgery is associated with higher although minimal risks (approximately 1%) [14] of internal organ injury (bowel, bladder and ureteric), hemorrhage, infection and mortality [15]. The treatment goal is to control the current episode of heavy bleeding and to reduce menstrual blood loss in subsequent cycles. The American College of Obstetricians and Gynecologists suggested that the selection of treatment for each woman depends on clinical stability, overall acuity, suspected etiology of bleeding, desired for future fertility and underlying medical problems [16].

## Clinical findings

### A. Brief overview of PALM-COEIN to determine etiology or association

Due to the confusing and inconsistent terms used to define abnormal uterine bleeding (AUB), the Federation Internationale de Gynecologie et d’Obstetrique (FIGO) has designed the PALM-COEIN classification system to define causes of AUB [17]. The component of PALM group includes structural causes; Polyp, Adenomyosis, Leiomyoma, Malignancy and COEIN group includes nonstructural causes: Coagulopathy, Ovulatory Disorders, Endometrial Disorders, Iatrogenic Causes, and Not Classified. The classification also defined intermenstrual bleeding (IMB) as it occurs between clearly defined cyclic and predictable menses while AUB was referred to as bleeding that is abnormal in volume, regularity and/or timing. HMB is a specific term, which is an abnormal volume of menstrual effluent and/or affecting the woman’s quality of life. Women with HMB would be defined as having AUB due to endometrial dysfunction (AUB-E) as many of them do not have identifiable structural or histological abnormality. However, other structural causes such as polyps (AUB-P), adenomyosis (AUB-A) and leiomyoma (AUB-L), as well as non-structural causes of coagulopathy (AUB-C) are also commonly related to HMB.

### B. Differential diagnosis for HMB from structural causes

Polyps, adenomyosis and leiomyoma are common structural abnormalities of the uterus, which are associated with abnormal bleeding. Despite the presence of these clinical findings, a thorough history and physical examination are needed to determine whether they are the cause of the abnormal bleeding. Location and size of uterine myoma (fibroids) are associated with varying amounts of menstrual blood loss. Myomas that increase the surface area of the endometrium such as sub mucosal myomas are more likely to be associated with bleeding abnormalities than myomas at other locations [18]. Intramural and cervical myomas are also associated with bleeding as they could distort the shape of endometrial surface, however subserosal myoma is less likely to be associated with abnormal bleeding. The mechanism(s) whereby myomas increase menstrual blood loss is unclear, but abnormal bleeding due to leiomyomas may be related to uterine vasculature abnormalities or impaired or dysregulated endometrial hemostasis [19].

Pelvic ultrasonography is an initial method to identify structural abnormalities related to bleeding both intracavity lesion and adnexal lesion such as arteriovenous malformation [20]. Saline infusion sonography could be used to further identify intracavity lesion such as endometrial polyp or sub mucosal myoma with high accuracy compared to hysteroscopy, however, hysteroscopy would be needed if biopsy or excision of the lesion is required [21].

### C. Differential diagnosis with disorders of hemostasis and coagulopathy

The coagulopathy (AUB-C) cause of HMB includes systemic disorders of hemostasis or coagulopathies. Von Willebrand disease is reported to be most common cause in this group that related to HMB with the prevalence of 13% [22]. Approximately 90% of AUB-C could be identified as a coagulopathy with use of the structured history screening criteria; 1.heavy menstrual bleeding since menarche; 2.one of the following symptoms: postpartum hemorrhage, bleeding related to a surgical procedure or bleeding associated with dental work; 3.two or more of the following symptom: bruising 1–2 times/month, epistaxis 1–2 times/month, frequent gum bleeding, or a family history of bleeding symptoms [23]. Other changes in hemostasis are rare and controversy exists over the findings as a cause of Heavy Menstrual Bleeding. Low normal Factor XI along with decreased platelet aggregation have been reported but normal values are variable and these test must be carried out in a center with expertise in their performance and standardization [24].

### D. Normal menstrual bleeding onset

A normal menstrual cycle has an average duration of menstrual bleeding of 4.5–8 days an interval of 24–38 days between the onsets of menses with 2–20 days of cycle to cycle variation over 12 months. The most blood loss occurs during the first 2 days of menstruation. The average blood loss with menstruation for normal women is ≤30 ml and menstrual blood loss more than 80 ml is considered abnormal [25]. The menstrual cycle variation in interval during puberty and early menarche and the perimenopausal transition is due to a high prevalence of anovulation and is probably not abnormal [26].

The endometrial functional layer undergoes characteristic changes of proliferation, secretion and degeneration reflecting the endogenous ovarian hormones. The endometrial basal layer is retained during menstruation and is the source of stem cells, glandular epithelial cells and stromal cells that regenerate the functional layer [27]. The epithelium (glandular and luminal epithelial cells) and mesenchyme (stroma and vasculature) also undergo morphologic changes during the cycle. Estradiol and progesterone withdrawal results in menstruation, which is endometrial breakdown with collagen degradation secondary to increased metalloproteinases, vascular disruption with bleeding and clot formation, cellular dissolution (apoptosis) and shedding associated with a local inflammatory process [28]. Cessation of menstruation may occur through morphological process of re-epithelialization of the luminal epithelium occurring without an increase of endogenous serum estradiol but a functional local process of vascular hemostasis and neoangiogenesis initiated and maintained by an increase in vascular endothelial growth factor (VEGF) [29–31].

### E. Multiple common pathways that contributes to HMB

Several different pathways that result or cause hemostatic dysfunctions have been implicated in increased menstrual blood loss [30]. The multiple pathways include the fibrinolytic system represented by plasminogen activator and its inhibitor, increased prostaglandinE2, local cytokines, and the influx of leukocytes into the endometrial stroma.

**Fig. 1:**
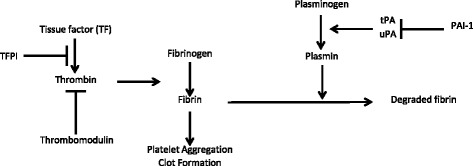
Tissue plasminogen activator (tPA) converts plasminogen to plasmin, which dissolve the fibrin in the blood clot. Plasminogen activator inhibitor-1 (PAI-1) and Tissue Factor (TF) are procoagulant by inhibiting plasminogen activators and increasing fibrin, respectively [34]. The figure is also separately submitted in a file name “figure1.jpg”

The fibrinolytic system consists of tissue plasminogen activator (tPA), and urokinase plasminogen activator (uPA) that are proteolytic enzymes, which are involved in lysis of the blood clot, local tissue remodeling and angiogenesis. The main enzyme is plasmin, which degrades fibrin into soluble degradation products thus lysing the intravascular clot. Both uPA and tPA convert plasminogen into plasmin. The activity of the plasminogen activators is regulated by plasminogen activator inhibitor-1 (PAI-1), which specifically binds tissue plasminogen activator [32–34] (See Fig. 1).

During the secretory phase of the cycle progesterone stimulates the expression of the procoagulant factors: Plasminogen activator inhibitor-1 (PAI-1) and tissue factor (TF). These procoagulant factors decrease with progesterone withdrawal. There is increased fibrinolytic activity with the onset of menstrual bleeding reflecting stromal dissolution and tissue and cellular breakdown [35, 36]. Women with HMB appear to have an increase of tPA, and reduction of PAI-1 resulting in increased collagenase and fibrinolytic activity [36–38]. This pathway has been demonstrated in endometrial endothelial cells as a possible mechanism that by enhancing clot integrity by decreasing tPA there could be less unscheduled spotting and bleeding in levonorgestrel intrauterine system users [39].

Progesterone withdrawal increases endometrial cyclooxygenase 2 (COX-2) enzyme, with a resultant synthesis and secretion of prostaglandin E2 (PGE2), and prostaglandin F2 alpha (PGF2a) [40]. Women with HMB have been demonstrated to have increased prostaglandin synthesis and COX-2 enzymes associated with heavy menstrual bleeding [33, 41, 42]. Prostaglandin E2 may contribute to excessive bleeding by enhancing vasodilatation of the spiral arteries, but there may be other mechanisms altering endothelial cell function and contributing to increased fibrinolysis.

Endometrial cytokines and metalloproteinases (MMPs) have been reported to be involved in HMB as they are initial mediators of the dissolution of endometrial collagen [30]. The proinflammatory cytokine tumor necrosis factor alpha (TNF-alpha) was significantly elevated and MMP-2 and MMP-9 were reduced in the menstrual effluent of heavy menstrual bleeders compared to women with normal bleeding [43]. During the late secretory phase, endometrial leukocyte infiltration occurs that up regulates tissue MMPs, which add to collagen breakdown and initiates the onset of menstruation [44–46]. There is no direct evidence for increased metalloproteinases in the endometrium of women with heavy menstrual bleeding.

No single pathway explains the cause of HMB. This has resulted in several therapeutic interventions specifically altering different pathways resulting in reduced blood loss with menstruation.

## Therapeutic interventions

### A. Combination hormonal contraceptives

Oral combination hormonal contraceptives (CHCs) reduce menstrual blood loss and result in a consistent menstrual cycle interval [47]. The reported reduction of MBL or PBAC score variously ranged from 32 to 69% at 3 months to 35–68% by 12 months [48]. CHCs could either be prescribed for 3 weeks, followed by 1 pill free week to allow withdrawal bleeding, or be given in extended cycle regimen to reduce number of withdrawal bleeding episodes, and the amount of blood loss, or continuous use of CHCs without the hormone-free interval to induce amenorrhea in 80–100% of women by 10–12 months [49, 50]. Although the reduction in MBL is generally assumed to be effective for all CHCs, the only formula that has been approved for therapeutic indication of HMB by both the European Union and the United States Food and Drug Administration is a combination of estradiol valerate and dienogest. A pooled analysis of randomized placebo controlled studies of this formulation showed that by treatment cycle 7, it significantly reduced median MBL by 88% compared to 24% with placebo [51]. The 1-year continuation rates of CHCs for treatment of HMB are 72–84%.

The possible but rare side effects of CHCs are breast tenderness, mood change, headache, nausea and vomiting. Contraindications to CHC use are women who are over 35 years old and smoke, have hypertension, cardio-vascular disease, migraine with aura, breast cancer, venous thromboembolism or thrombogenic mutation [52].

### B. Progestins

Progestin only regimens are safer alternatives for women with fewer contraindications compared to CHCs. Progestin only methods reduce MBL by inducing amenorrhea. Oral progestin only pills induced amenorrhea in 20% of women [53], but injectable medroxyprogesterone acetate (Depo-Provera) was reported to induce amenorrhea in 50% of women [54].

Oral norethindrone acetate (NETA) or medroxyprogesterone acetate (MPA) administer as short-course treatment (14 days per cycle) were reported to have limited efficacy in reducing MBL between 2 and 30%, but when administer as long-course (21 days per cycle) reduced MBL in 63–78% of the women [48]. Possible adverse effects for oral progestins are unscheduled bleeding, headache, breast tenderness, nausea and vomiting [52].

Intramuscular or subcutaneous injection of depot medroxyprogesterone acetate (DMPA) usually administered every 12 weeks induces amenorrhea by inhibition of follicular stimulating hormone thus inhibiting follicular development and reducing estradiol synthesis and secretion resulting in a thin endometrium and absent menstruation. Side effects of DMPA include unscheduled spotting and bleeding, weight gain, seborrhea of skin and hair, acne and bloating [52].

### C. Intrauterine system releasing levonorgestrel (LNG)

The levonorgestrel-releasing intrauterine system (LNG-IUS) initially releases 20 microgram of LNG per 24 h with a continuous local releases LNG that inhibits endometrial proliferation is associated with ovulation inhibition during the initial year after placement. LNG-IUS was approved for HMB treatment for up to 5 years in both the United Kingdom and United States. It is considered to be the most effective medical treatment for HMB as it induced a 70% reduction in MBL and PBAC scores during the first 3 months following insertion with a further reduction to 96% during the first year of use and continued efficacy for at least 4 years of treatment [48]. The 1-year continuation rate of LNG-IUS for treatment of HMB was 79%. As LNG-IUS locally releases the progestin, it has fewer side effects than the systemically administered progestins but a spontaneous expulsion rate of 7% is a drawback to its use [55]. Common side effects included unscheduled bleeding, breast tenderness, abdominal/pelvic pain/back pain, headache, ovarian cyst, and acne. The use of LNG-IUS is contraindicated in pregnancy, unexplained vaginal bleeding and uterine sepsis [52].

Among women with HMB and uterine structural pathology such as AUB-A (adenomyosis) and AUB-L (leiomyoma), LNG-IUS was also reported to be equally effective in MBL reduction and similar 1-year continuation rate [48] with rare uterine perforation (1:1000 cases) [52].

### D. Gonadotropin-releasing hormone agonists/antagonists

Gonadotropin-releasing hormone (GnRH) agonists and antagonists are synthetic decapeptides that bind to the GnRH receptor resulting in a decreased pituitary secretion of follicular stimulating hormone (FSH) and luteinizing hormone (LH). GnRH agonists initially cause a flare response, a rapid increase in FSH and LH, followed by desensitization of the receptor resulting in a hypogonadotropic hypogonadal state or pseudo menopause with low levels of FSH and LH. GnRH antagonist do not elicit a flare but immediately reduce FSH and LH secretion. Gonadotropin releasing hormone agonists suppress follicle development decrease ovarian hormone secretion and result in endometrial atrophy and amenorrhea [56]. Non peptide orally active Gonadotropin releasing hormone antagonists can also reduce heavy menstrual bleeding associated with uterine myomas but are not yet regulatory agency approved for clinical use [57].

GnRH agonists are approved by the Food and Drug Administration for patients with leiomyoma before the surgical interventions and have been used to treat endometriosis [58, 59]. The systematic reviewed concluded that GnRH agonists used for 3–4 months before leiomyoma surgery reduced menstrual blood loss and corrected preoperative iron deficiency anemia [60]. The endometrial atrophy usually occur within 3–4 weeks following initiation of treatment [61] with amenorrhea rate of up to 90% [62, 59]. However, there are several menopausal side effects such as vasomotor symptoms, vaginal atrophy, depression, and bone loss associated with their use [63]. The addition of estrogen and/or progestin therapy (add back therapy) is recommended in women who are symptomatic while taking the medication and to prevent bone loss due to hypoestrogenic state. The combination of GnRH agonists and low dose oral contraceptives (add back therapy) is reported to significantly decrease menstrual bleeding among women with HMB and increase the hematocrit level with minimal side effects [63].

### E. Tranexamic acid an anti-Fibrinolytic

Tranexamic acid is an anti-fibrinolytic drug that reduces blood loss given only with menstruation in women with HMB. Tranexamic acid significantly decreases endometrial tissue plasminogen activator activity, antigen and plasminogen activator inhibitor - type 1 antigen levels [64]. Tranexamic acid blocks the lysine binding site on plasminogen preventing its interaction with the lysine residues on fibrin. Plasmin is still formed from plasminogen but cannot degrade the fibrin (See Fig. 1) [34]. The recommended tranexamic acid dosage is 1 g orally 3–4 times daily during days of heavy bleeding. Tranexamic acid was reported to reduce MBL 34–56% with the use of >3 mg daily for 5 days and the greatest reduction in mean MBL was achieved in the first cycle of treatment [48]. If tranexamic acid does not decrease HMB within 2 cycles it should not be continued since the HMB is probably due to other causes. The side effects are gastrointestinal symptoms, headache, nausea and vomiting. As an anti-fibrinolytic agent, tranexamic acid could increase the risk for venous thromboembolism (VTE). Population-based studies have not found an association between tranexamic acid and increased VTE risk [65, 66]. Tranexamic acid should be used with caution in women with other risk factors for thrombosis or when prescribed with CHCs.

### F. Non-steroidal anti-inflammatory drugs

Inhibition of inflammatory mediators can help reduce the tissue damage at the time of menstruation. Non-steroidal anti-inflammatory drugs (NSAIDs) reduce the inflammatory process by inhibiting the cyclooxygenase enzymes that synthesize prostaglandins. The most commonly used NSAIDs for HMB are mefenamic acid, ibuprofen, naproxen, meclofenamate and flurbiprofen taken at the onset of menstruation. These drugs were reported to reduce MBL for 10–51% of women with HMB with a persisted effect up to 15 months [48]. Adverse effects are nausea, vomiting, abdominal pain and headache. NSAIDs are contraindicated in women with bleeding disorders or platelet function abnormalities because of their platelet aggregation properties and clotting factor enhancement [67].

### G. Danazol

Danazol is a synthetic androgen with weak androgenic biologic effects. Danazol inhibits FSH and LH secretion thus inhibiting follicle development with resultant endometrial atrophy. Taking orally, danazol was reported to reduce MBL by 80% in women with HMB [68–70]. Low dose vaginal danazol is proposed as an alternative treatment for HMB as it significantly reduce MBL and increase hematocrit, hemoglobin, and red blood cell count after 6 months of use with minimal adverse effects [71]. Data from a systematic review indicated that danazol is more effective in reducing MBL than placebo, progestogens, NSAIDs and the CHCs [72]. The side effects of danazol are androgenic effects such as hot flushes, myalgia, weight gain and acne, which occur in 85% of users [73, 74]. Although side effect of ovulation inhibition by suppressing hypothalamic-pituitary-ovarian axis was observed from oral danazol, it was not observed with vaginal danazol [71].

### H. Progesterone receptor modulators

Progesterone receptor modulators (PRMs) bind to the progesterone receptor and elicit tissue specific agonist, antagonist or mixed agonist/antagonist activity at the cellular level. PRMs alter the configuration of the progesterone receptor and result in endometrial morphologic changes suggesting an unopposed estrogenic effect with a relatively inactive appearing endometrium, low levels of mitotic activity and elevated incidence of apoptosis in the glandular epithelium [27]. Ulipristal acetate is the only PRM that is approved for clinical use for fibroids with HMB (Outside the United States). Ulipristal acetate effectively controlled HMB bleeding with a dose of 5–10 mg daily in 90% of women and induced amenorrhea in 70% of women [63–65]. Ulipristal acetate also reduced leiomyoma volume up to 50% with long term intermittent (3 months continuous use with a one month off) therapy [75–77].

Mifepristone 10 mg daily is reported to significantly reduced MBL by 95% and induce amenorrhea in 84% of women as well as increase hematocrit level during 3 months of treatment [78]. Mifipristone is not approved for treatment of HMB. Side effects of PRMs are minimal with headache and breast tenderness being the most common.

### I. Other options

There are several alternative therapies for women with HMB who have contraindications to the above therapeutic interventions [48]. These options include vasopressin analogues, hemostatic agents, selective estrogen receptor modulators, epsilon aminocaproic acid, gestrinone and interleukin 11. Desmopressin is a vasopressin analogue acting through vasoconstriction mechanism to reduce MBL. It has been shown to reduce median PBAC score by 24–42% during 2 cycles of treatment [79]. Ethamsylate is a hemostatic agent used to treat HMB given at 500 mg 4 times daily during days of menstruation it reduced MBL in 25% of women during 3 cycles of treatment and reduce MBL in 46% or women with 250 mg 4 times daily for 15 days per cycle [80, 81]. Ormeloxifene is a selective estrogen receptor modulator, which significantly inhibits endometrial proliferation and increase hematocrit level among HMB women. With a dose of 60 mg twice a week, it reduced PBAC scores up to 88% after 3 months of treatment with 9.5% of the women reporting amenorrhea. Epsilon aminocaproic acid, gestrinone and interleukin 11 are reported to reduce MBL 60–70% among HMB with coagulopathies or poor platelet aggregation [48]. The lack of a high level of efficacy of individual medical interventions reflects the multiple pathways involved in endometrial hemostasis. Desmopressin, Ethamsylate, and epsilon aminocaproic acid all have regulatory agency approval for use in bleeding disorders. There is no clinical evidence that these drugs reduce heavy menstrual bleeding.

A surgical intervention should be considered for women who are anemic due to heavy bleeding or have failed one or two medical treatments. The surgical treatment of HMB includes endometrial ablation, uterine artery embolization, hysterectomy and novel interventions such as laparoscopic bilateral uterine artery occlusion, transvaginal Doppler-guided vascular clamp, and laparoscopic and intrauterine ultrasound-guided radiofrequency ablation [82].

## Summary of treatments with recommendations

The American College of Obstetricians and Gynecologists proposed that medical treatment is the first line therapy for acute AUB women without systemic hematologic disorders, while surgical treatment would be considered based on stability of the patient, severity of bleeding, underlying disease, contraindications to medical treatment as well as lack of response to medical treatment [16]. Surgical treatment options ranges from in-office procedures to extensive surgery. The medical treatments include hormonal and non-hormonal options and the most effective in term of bleeding reduction are LNG-IUS, CHCs, tranexamic acid and long courses of oral progesterone [48].

## Conclusions

Although heavy menstrual bleeding is a common gynecological problem, there is a challenge in diagnosis and treatment as the condition cannot not be explained by a single hemostatic pathway. Several medical therapeutic options are available but each involves a different biologic pathway. Surgical intervention is always available but ablation of the endometrium or hysterectomy results in involuntary infertility and is associated with other morbidities. Thorough understanding of the clinical findings and the relationship to the bleeding pattern involving should lead to a tailored treatment for each individual patient.

## Abbreviations

AUB: Abnormal uterine bleeding
CHCs: Combination hormonal contraceptives
COX-2: Progesterone withdrawal increases endometrial cyclooxygenase 2
DMPA: Depot medroxyprogesterone acetate
FIGO: Federation Internationale de Gynecologie et d’Obstetrique
FSH: Follicular stimulating hormone
GnRH: Gonadotropin-releasing hormone
HMB: Heavy menstrual bleeding
IMB: Intermenstrual bleeding
LH: Luteinizing hormone
LNG: Levonorgestrel
LNG-IUS: Levonorgestrel-releasing intrauterine system
MBL: Menstrual blood loss
MMPs: Metalloproteinases
MPA: Medroxyprogesterone acetate
NETA: Oral norethindrone acetate
NSAIDs: Non-steroidal anti-inflammatory drugs
PA: Plasminogen activators
PAI-1: Plasminogen activator inhibitor-1
PBAC: Pictorial blood loss assessment chart
PGF2a: Prostaglandin E2 (PGE2), and prostaglandin F2 alpha
PRMs: Progesterone receptor modulators
TF: Tissue factor
TNF-alpha: Tumor necrosis factor alpha
tPA: Tissue-type plasminogen activator
uPA: Urokinase-type plasminogen activator
VEGF: Vascular endothelial growth factor
VTE: Venous thromboembolism
